# Biochemical, Kinetic and Biological Properties of Group V Phospholipase A2 from Dromedary

**DOI:** 10.3390/molecules27113437

**Published:** 2022-05-26

**Authors:** Mona Alonazi, Aida Karray, Raida Jallouli, Abir Ben Bacha

**Affiliations:** 1 Biochemistry Department, Science College, King Saud University, P.O. Box 22452, Riyadh 11495, Saudi Arabia; moalonazi@ksu.edu.sa; 2 Laboratoire de Biochimie et de Génie Enzymatique des Lipases, ENIS Route de Soukra, Université de Sfax-Tunisia, Sfax 3038, Tunisia; aida.karray@enis.tn; 3 Institut de Pharmacologie de Sherbrooke, Université de Sherbrooke, Sherbrooke, QC J1H 5N4, Canada; jallouliraida@yahoo.fr; 4 Laboratory of Plant Biotechnology Applied to Crop Improvement, Faculty of Science of Sfax, University of Sfax, Sfax 3038, Tunisia

**Keywords:** phospholipase V, kinetics, characterization, biological activities

## Abstract

Secretory group V phospholipase A2 (PLA_2_-V) is known to be involved in inflammatory processes in cellular studies, nevertheless, the biochemical and the enzymatic characteristics of this important enzyme have been unclear yet. We reported, as a first step towards understanding the biochemical properties, catalytic characteristics, antimicrobial and cytotoxic effects of this PLA_2_, the production of PLA_2_-V from dromedary. The obtained DrPLA_2_-V has an absolute requirement for Ca^2+^ and NaTDC for enzymatic activity with an optimum pH of 9 and temperature of 45 °C with phosphatidylethanolamine as a substrate. Kinetic parameters showed that *K_cat_*/*Km*_app_ is 2.6 ± 0.02 mM^−1^ s^−1^. The enzyme was found to display potent Gram-positive bactericidal activity (with IC50 values of about 5 µg/mL) and antifungal activity (with IC50 values of about 25 µg/mL)in vitro. However, the purified enzyme did not display a cytotoxic effect against cancer cells.

## 1. Introduction

Phospholipases A2 (PLA_2_) are a family of enzymes that hydrolyze the ester bond at the sn-2 position of phospholipids generating free fatty acids and lysophospholipids [1].

This family includes a number of secreted PLA_2_s (sPLA_2_s) referred to as group IB (GIB), GII (subgroups A–F), GIII, GV, GX and GXII (subgroups A–B) [2]. Clearly, the different mammalian sPLA_2_s are not isoforms, since only 15% of their primary sequences are identical [3,4,5]. They have distinct enzymatic properties [6,7] and show different tissue distribution patterns in both mice and humans. Consequently, in various tissues, the different sPLA_2_s may exert distinct biological functions that may be dependent or independent of their enzymatic activities [3,6,7]. In addition, in the same cell, the expression of the various isoforms may be differentially regulated by such events as differentiation or activation. Therefore, the profile of sPLA_2_s secreted in inflamed tissues can vary according to the type of inflammation and of infiltrating cells. Most sPLA_2_s are stored within inflammatory cells and are released in the extracellular environment upon appropriate cell activation [1,2]. Thus, large quantities of sPLA2s are released in plasma and biological fluids during local or systemic inflammation [8].

Group V sPLA_2_ has been cloned from chicken [9], human, rat, and mouse species [10]. Unlike group I and II sPLA2s, this sPLA_2_ has only six disulfides and does not have the group I- or group II-specific disulfides, thus defining a novel group of sPLA_2_s [11]. This sPLA_2_ has a higher level of identity with group IIA sPLA_2_s, as compared to group IB sPLA_2_. It neither has a propeptide sequence, indicating its closer relationship with group II sPLA_2_s. sPLA_2_ (group I/II/V/X) are closely related molecules with low molecular weight, 14–19 kDa, and possess very high structural conservation. All of these sPLA_2_s possess a Ca^2+^ binding loop and a catalytic dyad formed by His/Asp, as well as conserved disulfide bonds, while atypical sPLA_2_s (group III/XII) each form a distinct class [4,12,13,14]. In humans, the study of the structure–function relationship of sPLA_2_ isoforms is important for a better understanding of the pathology of diseases related to these enzymes. sPLA_2_ strictly hydrolyzes fatty acyl esters at the 2-position of the glycerophospholipid and exhibits substrate specificity in terms of polar or fatty acid headgroups at the sn-2 position [7]. For example, sPLA_2_-X is highly active on neutral phosphatidylcholine (PC), whereas sPLA_2_-IIA has much greater affinity for charged phospholipid head groups, especially phosphatidylserine (PS), phosphatidylglycerol (PG) and phosphatidylethanolamine (PE). This preference is useful for understanding the role of sPLA_2_-IIA as defensive proteins, acting on PE and PG, which are major components of bacterial membranes [15]. Mammalian sPLA_2_-V has a preferential expression level in the heart and a much lower expression level in lungs and liver [16]. In humans, the ability of sPLA_2_-V to regulate phagocytosis is specific and not shared with cytosolic PLA2 alpha (cPLA_2_α) nor sPLA_2_-IIA [17].

The common of sPLA_2_ isoforms are up-regulated by proinflammatory stimuli such as bacterial lipopolysaccharide (LPS), which largely increases the expression of sPLA_2_-V. Additionally, it has been shown that sPLA_2_-V is considered as a significant messenger in the regulation of cell migration. Indeed, Lapointe et al. [18] investigated the effect of sPLA_2_-V on LPS-mediated leukocyte recruitment supporting the involvement of sPLA_2_-V in the development of inflammatory innate immune response and its capacity to modulate adhesion molecule expression. Indeed, immunohistochemistry studies showed that sPLA_2_-V is expressed in the airways of patients with pneumonia but not those of normal individuals [19]. Moreover, it was shown that activated cells secrete sPLA_2_-V which exert transcellular lipolytic activity on neighbouring inflammatory cells [20]. The elevation of sPLA_2_-V expression in mice lungs with severe inflammation can be associated with an ongoing surfactant hydrolysis often observed in lung dysfunction [21]. Interestingly, sPLA2-V is involved in the innate immune response against bacteria and fungi: it is involved in the phagocytosis reaction and in lysis following a mechanism dependent on the fusion of phagosomes [17].

Until now, only a few studies were focused on the regulation and biological roles of phospholipases A_2_ from dromedary [22,23]. Accordingly, the present work was undertaken to further investigate the biochemical and antibacterial properties of dromedary non-digestive PLA_2_ to compare them with known PLA_2_-IIA and to gain further insights onto their mode of action with regard to phospholipids. This study also reports, for the first time, on the purification, characterization, and antibacterial activities of a novel PLA_2_-V secreted from the heart of dromedary

## 2. Results

### 2.1. Biochemical Properties of DrPLA_2_-V

As described previously, sPLA_2_-V has been shown to be principally implicated in the inflammatory processes [24], but the biochemical and enzymatic properties of this essential enzyme have been indistinct. In order to gain further insights onto its mode of action with regard to phospholipids, we reported the enzymatic catalysis and the biological functions of this PLA_2_, purified from dromedary heart tissue.

PLA_2_-V is purified and characterized from the dromedary heart (delipidated powder). The purification flow sheet presented in Table 1 showed that the specific activity of pure DrPLA_2_-V reached 115 U/mg when phosphatidylethanolamine (PE) was used as a substrate at pH 9 and 45 °C, in the presence of 8 mM NaDC and 4 mM CaCl_2_. The DrPLA2-V purification yield was around 44% of the total initial activity, a value which is comparable to what was observed with the dromedary, porcine and ostrich PLA_2_-IB [23,25].

**Figure 1:**
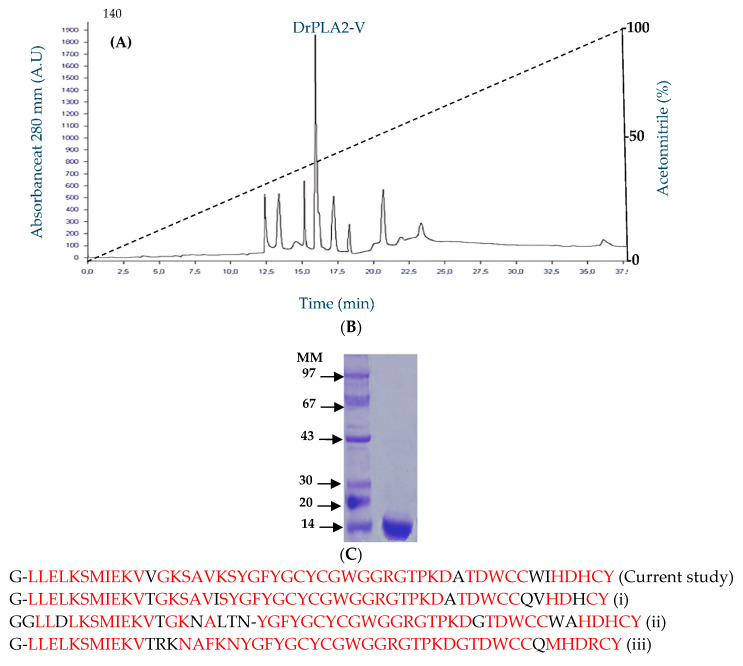
(**A**). Chromatography on RP-HPLC column of the purified DrPLA_2_-V from dromedary heart. RP-HPLC on a C18 column pre-equilibrated in solvent A, elution was performed using a gradient from 0% to 100% solvent B at a flow rate of 1 mL/min. Solvent A is composed of water/trifluoroacetic acid TFA (1000:1, *v*/*v*) and solvent B contained 100% acetonitrile. The gradient is indicated by the dotted line. The absorbance was measured at 280 nm. AU: Arbitrary Units. (**B**) 15%-SDS-PAGE of pure DrPLA_2_-V. Lane 1, molecular mass markers (kDa); lane 2, 10 µg of purified DrPLA_2_-V. (**C**) NH2 sequence alignment of DrPLA_2_-V, *Miniopteridae* family (*Miniopterusnatalensis*) (XP_016070213.1) (i), human family (*homosapiens*) (NP_000920.1) (ii), and *Hyaenida* family (***Hyaena hyaena***) (XP_039084994.1) (iii). Identical amino acids are shown in red.

The procedure described and summarized in Table 1 is more rapid than those used previously to purify another mammalian pancreatic phospholipase A_2_. In fact, the enzyme was purified after a heat treatment at 70 °C, and ammonium sulphate precipitation (20–65%), followed by only one chromatographic step (Figure 1A) whereas in the case of the dromedary, porcine or ostrich pancreatic PLA_2_ four chromatographic steps were needed [25]. The molecular mass of the purified enzyme was 14 kDa to secrete PLA_2_ (Figure 1B).

The NH2-terminal sequencing permitted clearly the detection of 44 residues of the pure enzyme: GLLELKSMIEKVVGKSAVKSYGFYGCYCGWGGRGTPKDATDWCCWIHDHCY. The N-terminal sequence alignment of sPLA_2_-V showed a high degree of homology with those of *Miniopteridae* family (*Miniopterusnatalensis*) (XP_016070213.1) [26], human family (*homosapiens*) (NP_000920.1) [27], and *Hyaenida* family (*Hyaena hyaena*) (XP_039084994.1) [28] of about 92%, 84% and 82%, respectively (Figure 1C).

**Figure 2:**
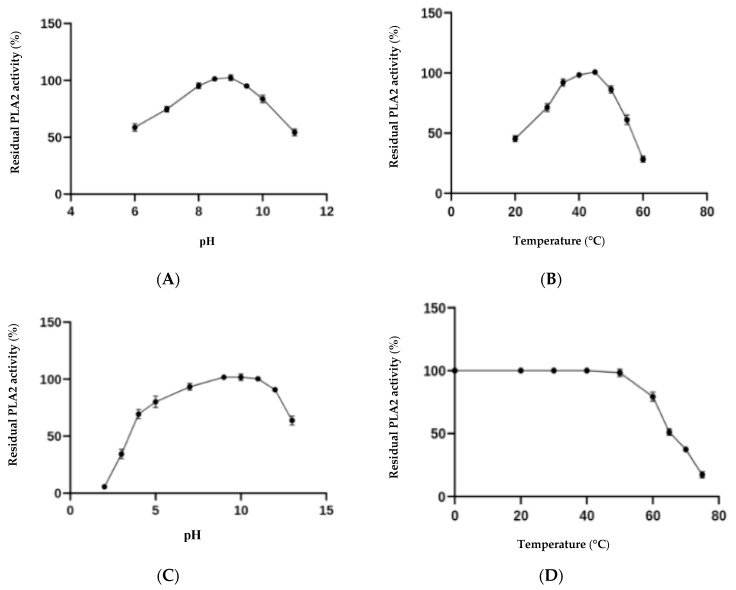
Evaluation of pH and temperature effect on activity (**A**,**B**) and stability (**C**,**D**) of DrPLA_2_-V.

The purified DrPLA_2_-V was found to be stable between pH 4.0 and 12.0. In contrast, the enzyme was found to lose almost its full activity when incubated at pH 2. It was also reported that dromedary [22], stingray [29], porcine [30] rat [31] and human [32] intestinal PLA_2_s are stable at low pH values. Unlike pancreatic DrPLA_2_-IB, which is completely denatured at high temperature, the DrPLA_2_-V maintained about 80% of its activity when incubated for 60 min at 60 °C (Figure 2). Similar observations were obtained previously with intestinal PLA_2_ from various mammal species showing high stability at elevated temperatures [33]. These results were obtained when we used the pH-stat method (with emulsified phosphatidylcholine (PC) as substrates).

**Figure 3:**
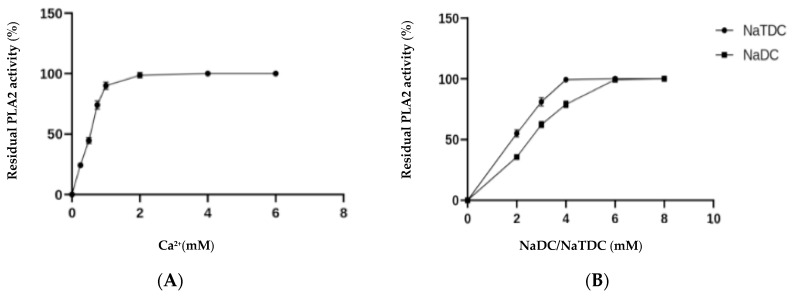
Effect of calcium ions (**A**), and surfactant (**B**), on DrPLA_2_-V activity. The incubation time with the appropriate agent was for a period of 60 min and the remaining phospholipase activity was evaluated at the optimal conditions.

As all secreted PLA_2_, the Ca^2+^ ions are essential for DrPLA_2_-V to express its full activity, with an optimum at 4 Mm (Figure 3A). All the divalent ions tested were unable to express the full specific activity of the enzyme. Figure 3B shows that both NaTDC and NaDC were required to express the maximal activity at concentrations of 4 and 6 mM, respectively.

**Figure 4:**
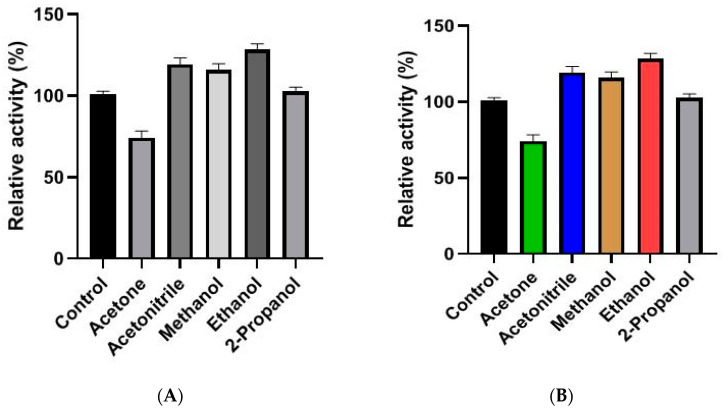
Effect of organic solvents on DrPLA_2_-Vstability. Enzyme was incubated with the appropriate agent for 1 h (**A**) and 2 h (**B**) and the remaining phospholipase activity was tested at the optimal conditions.

The purified PLA_2_ displayed better functional stability in the presence of polar solvents after an incubation time of 2 h, compared to the control test (Figure 4). It reaches 105% of its activity in presence of acetonitrile, 100% in the presence of methanol and 2-propanol and 124% in the presence of ethanol. In fact, it has been proved that organic solvents are advantageous in various industrial enzymatic processes since their use can increase the solubility of non-polar substrates, the thermal stability of enzymes, or eliminate microbial contamination [34].

### 2.2. Kinetic Parameters Determination of The PLA_2_-V from Dromedary Using Phospholipids (PL) of Different Head Groups

Then, the kinetic properties of DrPLA_2_-V (tested with three different phospholipids head groups) using the emulsified system were studied. The data obtained (summarized in Table 2) showed the clear capacity of DrPLA_2_-V to hydrolyze PE compared to DrPLA_2_-IB with *V_max_* value of 115 ± 3.5. The latest enzyme shows a clear preference for the zwitterionic substrate: PC. Less affinity was observed with PC with a catalytic constant value of 20.3 ± 0.7 compared to that obtained with PE (26.9 ± 1.2). Whereas, phosphatidylserine (PS) showed the lowest specific activity with a specific activity of 32 U/mg ± 1.2. This observation is confirmed by the activity of group V PLA_2_ from stingrays which hydrolyze PE (72 U/mg ± 1.5) and PC (52 U/mg ± 3.5) substrate more efficiently than PS substrate (18 U/mg ± 0.7) [35]. Besides, human heart sPLA2-V preferentially hydrolyzes PE vesicles compared to PC vesicles [36].

### 2.3. Bactericidal Properties, Antifungal and Cytotoxic Effect of Dromedary PLA_2_-V

The antimicrobial activity of the purified DrPLA_2_-V against Gram+ and Gram- bacteria was evaluated in the current study and its effectiveness was qualitatively and quantitatively determined (detection of the inhibition zones, IC50 and MIC values). Results are summarized in Table 3.

The inhibition zones were obtained only against Gram+ bacteria and ranged from 12 mm ± 0.5 (against *S. pyogenes* (ATCC 21059)) to 18 mm ± 0.7 (against *L. monocytogenes* (ATCC 19111) and *S. aureus* (ATCC 25923)). IC50 values were nearly the same 3–6 µg/mL. Gram- bacteria were resistant to the action of DrPLA_2_-V. The current enzyme is much more effective than marine group V-PLA_2_ showing IC50 values between15–25 µg/mL [37]. Both enzymes were inactive against Gram- bacteria. Interestingly, the antifungal effect observed in the present study shows IC50 values nearly above 25 µg/mL against all the tested strains. The enzyme was less effective against fungi than against Gram+ bacteria (Table 3).

Previously, it was reported that the antibacterial effect is strongly correlated with the enzymatic hydrolyze of the phospholipid bacterial cell membranes. The PLA_2_-V is able to break into the cell wall of Gram-positive bacteria [38]. Its efficiency to act against Gram-positive bacteria is basically affected by the charge of the overall cation on the surface of the enzyme molecule [39].

**Figure 5:**
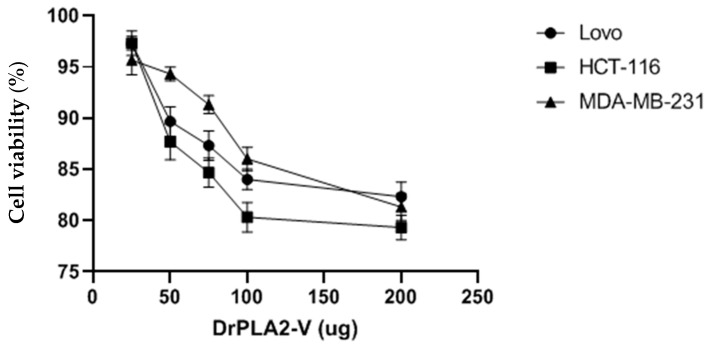
Cytotoxic potency of DrPL_2_ -V on Lovo, HCT-116, and MDA-MB-231 cells. Cytotoxicity was assessed using the MTT assay by incubating cells for 24 h with various concentrations (25, 50, 100, and 200 μg) of *DrPLA_2_-V*.

When we moved to the analysis of the cytotoxic effect of the dromedary V-PLA_2_ we noticed that the proportion of viable Lovo, HCT-116, or MDA-MB-231 cells in experimental conditions which contain 50 µg of group V, sPLA_2_, and calculated after treatment of 24-h-period, was constantly more than 85% (Figure 5). No difference was seen when we increased enzyme concentration to 200 µg of the pure enzyme. Thus, we can conclude that V-PLA2 is the noncytotoxic enzyme, like all sPLA_2_. This result is confirmed by [35].

## 3. Discussion

PLA_2_ catalyses the glycerophospholipids at the sn-2 position, generating free fatty. To date, the sPLA_2_ are classified into 10 catalytically active enzymes in mammals, and are characterized bylow-molecular-weight and Ca^2+^-requiring extracellular enzymes. Each sPLA_2_ showed a distinctive expression profile in all cell types within restricted tissues. As described previously in cellular studies, sPLA2-V revealed their involvement in the inflammatory processes [24], but the biochemical and enzymatic properties of this current enzyme have been poorly documented until now. We report, as the first step towards understanding the structure, function and regulation of this PLA_2_, the production and characterization of DrPLA_2_-V. Evaluation of the antimicrobial effect of the enzyme and its cytotoxicity is also studied.

Unlike PLA_2_-IB and PLA_2_-X, characterized with the presence of a propeptide cleaved by an endogen trypsin in order to produce a mature and active enzyme, we reported here that no significant increase in the DrPLA_2_-V activities was observed throughout 1 h homogenization with endogenous trypsin (data not shown). Moreover, it was noted that PLA_2_-V lost its full activity after an addition of trypsin at a final concentration of 20 g/mL. These observations permit to suggest that an accessible site of trypsin cleavage is present in the PLA_2_-V primary sequence. A total of 50 g of dromedary heart mucosa (6 U/g of heart tissue) was obtained using 50 mL of 25 mM Tris–HCl pH 8 with 4 mM benzamidine and 150 mM NaCl. The purification steps consist of a heat treatment for 10 min at 70 °C, followed by sulphate fractionation (20–65%). The obtained precipitate is dialyzed against the same buffer after repeated changes and then loaded onto C18 HPLC column pre-equilibrated with 0.1% TFA in water and then eluted with an acetonitrile linear gradient 0–80%. After the purification procedure of the PLA_2_, the analyzed fractions on SDS-PAGE indicate that the current enzyme (named DrPLA_2_-V) presents an apparent molecular mass of about 14 kDa. The specific activity of pure DrPLA_2_-V reaches 115 U·mg^−1^ when PE was used as substrate at pH 9, 45 °C and in the presence of 4 mM CaCl_2_ and 8 mM NaDC, a value which is comparable to that observed with the PLA_2_-V from chicken or stingray with a specific activity of 156 or 52 U/mg, respectively, measured on the same substrate [9]. The DrPLA_2_-V purification yield was about 44% the total initial activity (Table 1).

N-terminal sequence of DrPLA_2_ showed a high level of identity with those of the sPLA_2_-V from other species. The purified enzyme showed pH stability between pH 4.0 and 12.0 and maintained about 80% of its activity after 60 min of incubation at 60 °C. Comparable results were obtained previously with mammalian PLA_2_-Vfrom various species showing a good stability at high temperature [40] Moreover, CaCl_2_ (4 mM) was found as the best activator of the PLA_2_ activity of pure *DrPLA_2_-V*, followed by Mg^2+^ (combined with 1 Mm CaCl_2_). All crystal structures of sPLA2 have a ‘calcium binding loop’ in the protein [41] and the calcium dependence of the group V PLA2 is similar to that of the human group IIA PLA_2_ [24].

We next studied the kinetic properties of DrPLA_2_-V *Km*_app_, *K_cat_* and the deduced catalytic efficiency (*K_cat_*/*Km*_app_) of the purified group-V, using charged PE, zweeterionic PC or PS as substrate using Lineweaver–Burk plots. The data obtained (Table 2) showed the clear capacity of DrPLA_2_-V to hydrolyze the negatively charged substrate PE (*Km*_app_ 115 ± 3.5) compared to the zwitterionic substrate PC (*Km*_app_ 87 ± 2.1) and PS (*Km*_app_ 32 ± 1.2). Our results clearly demonstrated that the enzyme hydrolyzes PE and PC substrate more efficiently than PS substrate since it presented a catalytic efficiency (*K_cat_*/*Km*_app_) eight or five times higher than those obtained with using PE as substrates. The same trend was observed using PC or PE as a substrate. This result is in line with Chen and Dennis [36] who have also demonstrated that human heart sPLA2-V preferentially hydrolyzes PE vesicles compared to PC vesicles. Likewise, ref. [35] reported that stingray PLA2-V hydrolyses the zwitterionic PE and PC substrates more efficiently than anionic PS substrate.

Furthermore, proinflammatory stimuli such as bacterial LPS, cause an up-regulation of the majority of sPLA_2_ isoforms, and thus predominantly increase the expression of sPLA2-V. Besides, it has been recently shown that sPLA_2_-V is a critical messenger in the regulation of cell migration and has a specific function related to phagocytosis [17]. In the current study, we have demonstrated a very effective Gram-positive bactericidal activity for DrPLA_2_-V, producing an inhibition zone of 18 mm against *B. cereus*, *S. aureus* and *L. monocytogenes* compared to the control Ampicillin producing an inhibition zone ranging from 20 to 26 mm. Contrary, Dr PLA_2_V was inactive against *E. coli* and against Gram^−^ bacteria. Besides, the antifungal effect of the purified enzyme is attributed to its phagocytosis role against pathogenic strains: bacteria and fungi. Previous results showed that macrophages from sPLA_2_-V^−/−^ mice stimulated with zymosan (a complex of proteins and carbohydrates extracted from the membrane of yeast cells) produced 50% less leukotriene C4 and prostaglandin 2 than normal mouse macrophages, and also show a 50% reduction in their phagocytic capacity [17]. As a result, sPLA_2_-V is involved in the innate immune response against fungi: it is involved in the phagocytosis reaction and in lysis following a mechanism dependent on phagosome fusion. However, to date, the regulation of fungal phagocytosis by sPLA_2_-V is not yet well-detailed. In fact, we showed here a positive effect against all the tested fungi strains. Whereas, the current study indicated that DrPLA2-V did not affect any lines of human cancer cells (HCT-116, MDA-MB-231 and Lovo) [42] These results are in lines with all secreted PLA_2_ tested on normal and cancer cells, suggesting that all sPLA_2_s are noncytotoxic enzymes.

## 4. Materials and Methods

### 4.1. Phospholipase Activity and Protein Concentration Determination

Phospholipase activity was measured titrimetrically according to Abousalham and Verger [43] with a pH-stat using a crude egg yolk, PC, PE or PS emulsions as a substrate in the presence of 8 mM NaDC and 4 mM CaCl_2_ at optimal conditions (pH 9 and at 45 °C). A total of 1 µmol of fatty acid released per minute is equivalent to one unit of phospholipase activity. Protein content was determined according to the Bradford (1976) method [44] using bovine serum albumin (*E*^1%^_1cm_ = 6.7) as a reference.

### 4.2. Group V DrPLA_2_ Purification

Heart collection and phospholipase homogenization: Fresh heart tissue of dromedary was collected immediately after slaughter (Riyadh, Saudi Arabia) and kept at −20 °C. The soluble extract obtained from 50 g of dromedary heart mucosa (6 U/g of heart tissue) was obtained using 50 mL of 25 mM Tris–HCl pH 8 with 4 mM benzamidine and150 mM NaCl followed by a centrifugation at 25,600× *g* during 20 min.

Heat treatment: The homogenate (300 U) was incubated for 10 min at 70 °C, rapidly cooled, and then centrifuged during 40 min at 25,600× *g*.

Ammonium sulphate precipitation: The clear supernatant obtained containing 78.7% (236 U) of the initial activity was subjected to ammonium sulphate fractionation (20–65%). The precipitates were resuspended in the extraction buffer and dialyzed against repeated changes in the same buffer (after 4, 8 and 12 h) for 24 h at 4 °C.

C-18-HPLC chromatography: Thereafter, the dialyzed sample was loaded on a C18 HPLC column (250 × 4.6 mm, 5 mm; Beckman, Fullerton, CA, USA) pre-equilibrated with 0.1‰ TFA in water and then eluted with an acetonitrile linear gradient 0–80% at a flow rate of 1 mL/min over 60 min. The active fractions were analyzed with 15%-SDS-PAGE according to Laemmli [45], while the PLA_2_ activity was monitored as described above. The N-terminal sequence was determined automatically with Edman’s degradation, using an Applied Biosystems Protein Sequencer Procise 492 equipped with 140 C HPLC system [46].

## 5. Biochemical Properties

### 5.1. pH and Thermal Activity and Stability of DrPLA_2_

The pH and thermal activity of phospholipase were measured on a crude egg yolk emulsion as substrate at pH values (6–11) or temperatures (20 to 60 °C), respectively.

Additionally, the pH and thermal stability were measured at extreme pH and temperature values by incubating the same amount of pure enzyme at different pH (2–13) or temperature values (20–70 °C) for 1 h, respectively. The residual activity was determined under standard assay conditions.

### 5.2. Effect of Metal Ions and Surfactant (NaDC/NaTDC) on DrPLA_2_-Vactivity

The hydrolysis rates of the PC egg yolk emulsion by PLA_2_ were measured in the presence of Ca^2+^ at different concentrations from 0 to 10 mM at pH 9 and at 45 °C while the effects of 10 mM divalent metal ions (Cd^2+^, Mg^2+^, Mn^2+^, or Zn^2+^) on the enzyme activity were evaluated with the presence of 1 mM Ca^2+^.

Furthermore, the rate of hydrolysis of PC by DrPLA_2_-V with concentrations ranging from 0 to 10 mM of natural surfactant NaDC or NaTDC, at pH 9 and at 45 °C was also studied.

### 5.3. Effect of Organic Solvents on sPLA_2_ Stability

The effect of organic solvents on sPLA_2_ stability was determined after incubation of the enzyme in the presence of acetone, acetonitrile, methanol, ethanol and 2-propanol (50%, *v*/*v*) at 25 °C for 1 and 2 h. The residual activity was calculated and compared to the control, after centrifugation for 5 min at 13,500× *g*, at pH 9 and at 45 °C.

### 5.4. Kinetic Parameters

The activity of the purified enzymes was evaluated at various final concentrations ranging from 0 to 60 mM of PC, PE and PS under optimal conditions (pH 9, 45 °C and in the presence of 4 mM CaCl_2_ and 4 mM NaTDC). Measurements were recorded in duplicate and the respective kinetic parameters, including *V_max_* and *Km*_app_ were calculated from Lineweaver–Burk plots (Lineweaver and Burk, 1934). The turnover number (*K_cat_*) value was determined from the following equation: *K_cat_* = *V_max_*/[E], where *V_max_* is the maximal velocity and [E] is the active enzyme concentration.

## 6. Antimicrobial Activity

Pure standard microbial isolates collected from King Khaled University Hospital were tested in this study; including four fungal strains (*P. digitatum*) and 12 bacterial strains: *Escherichia coli* (*E. coli*; *ATCC 25966*), *pseudomonas aeruginosa* (*P. aeruginosa*; *ATCC 27853*), *Enterobacter aerogenes* (*E. aerogenes*; *ATCC 13048*), *and Salmonella enterica* (*S. enteric ATCC*; *43972*) as Gram-negative, *Bacillus cereus* (*B. cereus*; *ATCC 14579*), *Bacillus subtilis* (*B. subtilis*; *ATCC 6633*), *L. monocytogenes* (*ATCC 19111*), *Enterococcus faecium* (*E. faecium*; *ATCC 19433*), *Streptococcus pyogenes* (*S. pyogenes*; *ATCC 21059*), *S. aureus* (*ATCC 25923*), *Staphylococcus epidermidis* (*S. epidermidis*; *ATCC 14990*), *Staphylococcus xylosus* (*S. xylosus*, *ATCC 700404*) as Gram-positive.

The agar diffusion method was performed to check the antibacterial activities of dromedary PLA_2_s. Bacteria were grown to mid-log-phase (OD600 = 0.8) in BHI medium. Fresh cultures (10 µL) of each microorganism were grown on 8 mL nutrient agar plates (Oxoid, UK); for bacterial suspension preparation of 0.5 MacFarland, containing 0.7% agar and poured over a 90 mm Petri dish containing 25 mL of 1.5% agar in BHI. Bacterial viability was investigated by determining the colony-forming ability (CFU) of bacteria incubated at different time intervals without or with appropriate amounts of the compound that was mixed with 2 × 10^7^ CFU/mL in sterile BHI and were incubated under shaking for 60 min at 37 °C. Samples were serially diluted into sterile BHI, streaked onto media agar plates, and incubated for 24 h at 37 °C. The antibacterial potency of tested compounds was expressed as the residual number of CFU with reference to the initial inoculums. Results presented as the half-maximal (50%) inhibitory concentration (IC_50_) are means of 3 different measurements. Additionally, the micro-well dilution method was used to determine the lowest compound concentration (MIC) that totally blocks the growth of tested microorganisms. Dilution series of the tested enzyme (10–200 µg/mL) were set in a 96-well plate. In each well, the mixture consisting of 50 µL of the diluted compound, 10 µL inoculums, and 40 µL of growth medium was incubated for 24 h at 37 °C. Then, 40 µL of MTT (0.5 mg/mL) was added to each well and the plate was again incubated for 30 min at the same temperature. The well showing no change to a violet-colored formazan compound indicates that the bacteria were biologically inactive and corresponds to the MIC. Ampicillin (1 mg/mL) was used as positive standard reference.

The disc diffusion technique using Sabouraud dextrose agar was employed to evaluate the antifungal activity of pure DrPLA_2_-V on some fungal strains (Ronald, 1991). Ten µg of the enzyme or the commercial cycloheximide (1 mg/mL), used as the positive control, was deposited on sterile paper discs that were placed then in the center of the inoculated Petri dishes and incubated at 30 °C for 24 h.

## 7. Cell Culture

Investigation of the cytotoxicity of the studied enzyme was carried out on human breast adenocarcinoma (MDA-MB-231) and colon cancer (HCT-116 and Lovo) cell lines (American Type Culture Collection; USA) using various amounts (25, 50, 75, 100, and 200 µg) of purified PLA_2_. Samples were first diluted in Dulbecco’s Modified Eagles Medium with 10% Fetal Bovine Serum, then added to cells grown and cultured in a 5% CO_2_-humidified incubator at 37 °C for 24 h. Thereafter, an ELISA end-point assay (Benchmark Plus, Bio-Rad, CA, USA) was performed to determine the activity of lactate dehydrogenase released from damaged cells in the collected supernatant aliquots. Negative and positive controls were in the assay medium only and 0.1% Triton X-100 in the assay medium, respectively. Cell viability, expressed as a relative percentage of the OD values (at 550 nm) for DrPLA_2_-V treated cells and the control, is shown as mean ± SD (n = 3).

## 8. Conclusions

The capacity of the dromedary to live under desert conditions and to survive in an incredibly harsh environment is due to its biological and physiological particularities. In the current study, some biological effects of PLA_2_-V from dromedaryare reported. The purified enzyme was biochemically characterized as pH- and temperature-stable, and the kinetic parameters were investigated (*K_m_*/*V_m_*/*K_cat_*). Our results indicate that the purified DrPLA_2_ is a potential enzyme candidate with therapeutic importance in pharmaceutical industry applications due to its antibacterial, and antifungal potential. Whereas, no cytotoxic effect was observed even at high concentrations tested. Thus, dromedaryis an efficient source of enzymes with high potential in biotechnological applications.

## Figures and Tables

**Table 1:** Flow sheet of DrPLA_2_-V purification.

Purification Step	Total Activity (Units)	Protein (mg)	Specific Activity (U/mg)	Activity Recovery (%)	Purification Factor
Extraction	300	2950	0.1	100	1
Heat treatment at 70 °C for 10 min	236	73.7	3.2	78.7	32
(NH_4_)_2_SO_4_ Precipitation (20–65%)	177	11.8	15	59	150
RP-HPLC	132	1.15	115	44	1150

1 Unit: μmole of fatty acid released per min using phosphatidylethanolamine as a substrate in the presence of 8 mM NaDC and 4 mM CaCl_2_.

**Table 2:** Apparent kinetic parameters of DrPLA_2_-V.

	*V_max_* (U/mg)	*Km*_app_ (mM)	*K_cat_* (s^−1^)	*K_cat_*/*Km*_app_ (mM^−1^ s^−1^)
PE	115 ± 3.5	10.5 ± 0.7	26.9 ± 1.2	2.6 ± 0.02
PC	87 ± 2.1	12.7 ± 0.3	20.3 ± 0.7	1.6 ± 0.03
PS	32 ± 1.2	21.3 ± 1.1	7.5 ± 0.3	0.3 ± 0.01

**Table 3:** Antimicrobial activity of DrPLA_2_-V on bacterial and fungal strains.

Strains	Inhibition Zone (mm)		IC50 (µg/mL)	MIC (µg/mL)
Gram (+) Bacteria	DrPLA2-V	Ampicillin/ Cycloheximide		
*B. cereus* (ATCC 14579)	18 ± 1	22 ± 1	6	>12
*B. subtilis* (ATCC 6633)	15.4 ± 0.6	25 ± 1	3.6	>6
*L. monocytogenes* (ATCC 19111)	18. 7 ± 0.5	21 ± 10.7	3.2	>6
*E. faecium* (ATCC 19433)	14.3 ± 1.2	18.5 ± 0.3	4.9	>9
*S. pyogenes* (ATCC 21059)	12 ± 0.5	15.5 ± 0.2	6.1	>15
*S. aureus*(ATCC 25923)	18 ± 0.7	21.5 ± 1.4	5.2	>12
*S. epidermidis*(ATCC 14990)	15.3 ± 0.6	26 ± 0.5	3	>9
*S. xylosus* (ATCC 700404)	16.9 ± 1.3	24 ± 1.2	2.9	>6
***Gram*** (***−***) ***Bacteria***				
*E. coli* (ATCC 25966)	*-*	22.6 ± 1.5	-	-
*P. aeruginosa* (ATCC 27853)	*-*	20 ± 0.7	-	-
*E. aerogenes* (ATCC 13048)	*-*	25 ± 1.2	-	-
*S. enteric* (ATCC 43972)	*-*	19.5 ± 0.3	-	-
***Fungi***				
*A. niger*	11.2 ± 0.3	28 ± 0.6	21 ± 1.5	>75
*B. cinerea*	9 ± 0.1	29 ± 1	31.7 ± 2.4	>90
*F. solani*	15 ± 0.7	27.5 ± 0.7	25 ± 2.1	>60
*P. digitatum*	7 ± 0.2	21 ± 0.5	35 ± 3.5	>90

## Data Availability

Not applicable.

## Sample Availability

Samples of the compounds are available from the authors.
